# Feature Attribution Analysis to Quantify the Impact of Oceanographic and Maneuverability Factors on Vessel Shaft Power Using Explainable Tree-Based Model

**DOI:** 10.3390/s23031072

**Published:** 2023-01-17

**Authors:** Donghyun Kim, Melia Putri Handayani, Sangbong Lee, Jihwan Lee

**Affiliations:** 1 Korea Marine Equipment Research Institute, Busan 49111, Republic of Korea; 2 Department of Industrial and Data Engineering, Major in Industrial Data Science and Engineering, Pukyong National University, Busan 48513, Republic of Korea; 3 Lab021 Shipping Analytics, Busan 48508, Republic of Korea

**Keywords:** explainable artificial intelligence, Shapley Additive exPlanations, TreeExplainer, vessel shaft power, regression, machine learning, maritime engineering, oceanographic, noon report

## Abstract

A vessel sails above the ocean against sea resistance, such as waves, wind, and currents on the ocean surface. Concerning the energy efficiency issue in the marine ecosystem, assigning the right magnitude of shaft power to the propeller system that is needed to move the ship during its operations can be a contributive study. To provide both desired maneuverability and economic factors related to the vessel’s functionality, this research studied the shaft power utilization of a factual vessel operational data of a general cargo ship recorded during 16 months of voyage. A machine learning-based prediction model that is developed using Random Forest Regressor achieved a 0.95 coefficient of determination considering the oceanographic factors and additional maneuver settings from the noon report data as the model’s predictors. To better understand the learning process of the prediction model, this study specifically implemented the SHapley Additive exPlanations (SHAP) method to disclose the contribution of each predictor to the prediction results. The individualized attributions of each important feature affecting the prediction results are presented.

## 1. Introduction

Marine transport is believed to be the primary medium of transportation for worldwide trade due to its efficiency of load. A major part of a whole cluster in global economic activities was carried on the ocean [1], but this comes with the contribution to the total greenhouse emission. International Maritime Organization (IMO) published its Fourth IMO Greenhouse Gas (GhG) Study in 2020 and stated that global shipping generated 1056 million tons of CO_2_ in 2018, which was equivalent to about 2.89% of all anthropogenic CO_2_ emissions globally. Additionally, using a voyage-based allocation method, international shipping contributed 740 million tons of CO_2_ to global shipping in 2018 [2].

Because global climate change is driven by the excessive production of pollution emissions, the shipping industry, which is the world’s largest transport sector, must place a high priority on lowering its emissions [3]. For the purpose of promoting energy efficiency, there has been a significant increase in research over the past few years on how to estimate the ship’s propulsion power.

Marine propulsion power can be examined by measuring the shaft power generated by the engine and it provides insight into how well the system is functioning [4]. Integrated systems that record the performance data of every element of a vessel engine have been widely implemented in the field of maritime engineering and it has made it set in motion of multiple research initiatives to conduct various studies by analyzing these data [5]. The research on vessel shaft power prediction took a major proportion of the many studies utilizing such data, and it has shown consistent improvement in recent years.

From 1977 until 1984, four research conducted by Holtrop [6,7] and Mennen [8,9], were carried out to develop the numerical description of a ship’s propulsion power properties and the related measurement. The regression-based method was implemented for each of the research, and it is believed to be one of the major initiatives for developing a prediction model of vessel shaft power. The use of various regression-based analyses in forecasting ship propulsion power has grown significantly during the last several decades. Along with the development of machine learning methodology, numerous research that aimed to predict vessel shaft power also made use of the progression of this advancement by utilizing each of the recent algorithms when the research was conducted.

Traced back to the past decade, there was some research deploying machine learning models to predict the shaft power or vessel propulsion power. In 2011 [10], two statistical investigations of ship energy efficiency analyzing the data collected from a domestic ferry were conducted by comparing the Gaussian process (GP) and Artificial Neural Networks. ANN resulted in slightly better performance than GP. Hence the continuation of the research in [11] that applied ANN in both real-time and predictive propulsion settings. There were some findings that ANN was indeed widely used with the aim of predicting shaft power. In 2014, data collected from the towboat were analyzed using ANN that was compared with an ensemble neural network (ENN) [12]. However, later, different machine learning models were showing some advancement. Thus, in 2017, a prediction of shaft power along with the prediction of shaft torque and fuel consumption were conducted by deploying a collection of machine learning techniques, including multiple Linear Regression (LR), LASSO regression, and Random Forest (RF) [13], which was categorized as the black-box model. Other than that, shaft power or vessel propulsion power-related research has been conducted using support vector-based machine learning models. Among those are Support Vector Machine (SVM) [14] and Support Vector Regression (SVR) [15], both in 2021. Both showed satisfactory results. Then, in 2021, another research also attempted to create a benchmark study by comparing multiple machine learning models, including eXtreme Gradient Boosting (XGBoost), Support Vector Machine, Linear Regression (MLR), Polynomial Regression, Generative Additive Model (GAM), Neural Networks, SVM, and a decision tree-based algorithm, XGBoost, which was the best-performing model in terms of error rate, as well as prediction accuracy (R-squared) [16]. A comparative study of the machine learning method to predict vessel shaft power was also conducted by [17], evaluating five models, which are MLR, AdaBoost, K-Nearest Neighbors (KNN), ANN, and RF, which resulted in RF as the best model.

Subsequently, before the recent period, numerous research has proved the optimum usage of machine learning algorithms to predict shaft power. However, those studies only experimented with the development of a prediction model without explainability. Thus, there has been no further in-depth investigation of how the machine learning system arrives at its prediction findings or how the many predictors that were considered throughout the process of developing the model influence the outcome of the prediction.

Numerous studies utilizing machine learning-based methodologies have been expanded with the aided Explainable Artificial Intelligence (XAI) approach to assist in better decision-making processes. This approach is making its way into a wide variety of domains, including education [18]; lithology [19] and geology [20]; social science [21]; construction engineering [22,23]; transportation [24] and smart cities [25]; healthcare [26] and medical [27]; mass media and entertainment [28]; tourism, travel, and hospitality [29]; supply chain management and manufacturing [30]; law enforcement [31] and legal [32]; information technology [33]; and financial services [34,35]. Overall, the research utilizing XAI to explain their machine learning model stated that it provides transparency of how the machine learning model produces its decision. To the best of our knowledge, however, in the maritime domain, we might be among the first to adopt the XAI methodology. One previous research pioneered the implementation of XAI in the maritime domain conducted anomaly detection analysis [36]. Thus, we are motivated to propose an extended framework for shaft power prediction using the XAI approach.

The objective of this research is to provide an interpretable explanation of the vessel shaft power prediction model using explainable AI techniques. This research not only contributes to the development of an explainable machine learning model for shaft power prediction but also goes deeper into the individualized attribution of each factor affecting the magnitude of shaft power using Shapley values. Factual oceanographic factors including tidal current, wind, wave, sea salinity, and temperature, as well as operating factors, including vessel speed, the rudder angle, the ship heading, and the drafts, were among the predictors for this analysis. In addition to that, the analysis was not only done over entire the dataset but also considering each distinct voyage number to understand the dynamic changes of feature importance with respect to different vessel trips.

To build an interpretable prediction model, we have carefully chosen input variables so that only oceanographic factors and operating factors are included in the prediction model. Oceanographic factors include wave, current, and wind that are externally given but may have a significant effect on the propulsion power. Operating factors, including speed, draft, and cargo weight, are directly controlled by the operator. In addition to this, most of the previous research has included intermediate sensor values that can be observed from the sensors at the vessel machinery sensor (such as RPM, piston, or cooling system) as their predictors. Although including these intermediate variables may increase the model’s accuracy, it is not good for explaining to the model because these intermediate variables are also the results of the vessel’s operation and not the direct cause of the shaft power. Thus, in this study, all the intermediate variables are excluded from the input variable. The experimental results, however, confirm that the model trained without intermediate variables could achieve a quite good prediction accuracy.

After then, we tried to provide a clearer explanation of the model using an explainable AI method. Focused on the oceanographic factors and the operating factors as the predictors of the prediction model, this research aims to specifically reveal which among those factors take hold of the generation of shaft power by the vessel propeller. More importantly, it is widely known that recent developments in machine learning and deep learning algorithms have made it possible and uncomplicated to construct any model with high-performance metrics. These developments have been widely publicized in numerous mediums. However, this will not be sufficient if the results cannot be confirmed or validated in the open; to put it another way, the “black box” that represents the model itself needs to be opened to discover how it reached the predicted results. By then, even the specific attribution of a single feature can be explained in terms of how it influences the outcome of the prediction.

Thus, the goal of this research can be presented by answering these three questions:

1. The vessel sails against sea resistance by adjusting its engine operation which generates the shaft power to drive the propeller. Is it possible to predict the vessel shaft power considering the influence of uncontrollable variables such as the oceanographic factors and also the controllable variables such as the maneuverability factors?
2. Among the uncontrollable and controllable variables affecting the generation of vessel shaft power, what factors deploy the significant influence and how?
3. Does a different vessel voyage (trip) change the important factors affecting the vessel shaft power?

## 2. Data Description

**Figure 1:**
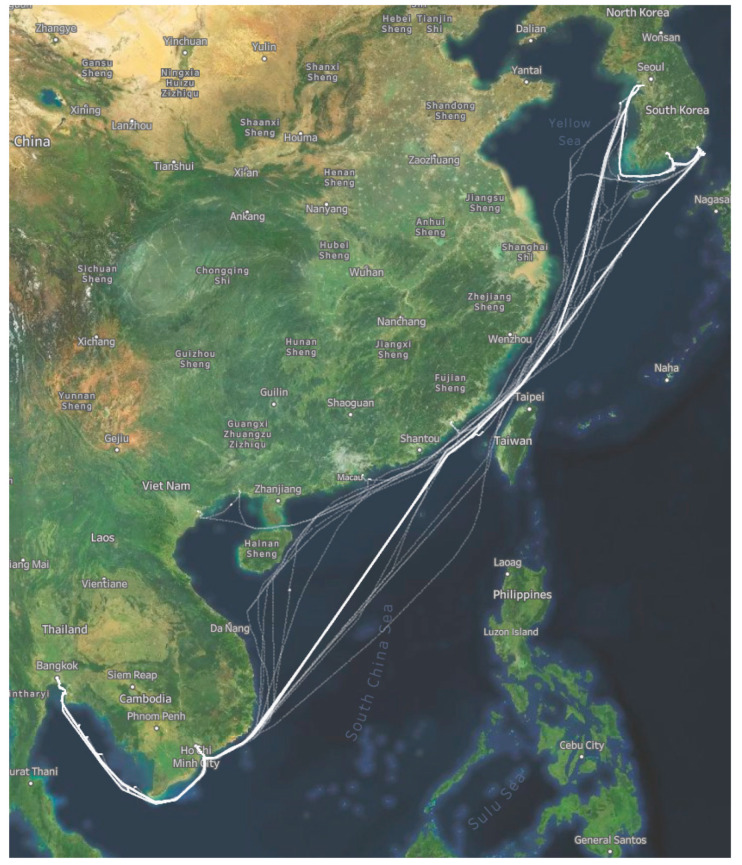
Vessel voyage routes.

This research utilized large datasets collected from vessel engine sensor data and the factual noon report of a 16-months voyage (January 2020 until June 2021) by a general cargo vessel with detailed specifications in Table 1. This vessel sailed for 24 different voyages crossing some Asian countries such as South Korea, China, Taiwan, Vietnam, and Thailand. The routes of the whole voyage can be seen in Figure 1.

In general, the sensor data consists of automated system records of all sensors installed in the vessel engine, and this system is used to control the performance of each of the engine parts. On the other hand, the day-to-day operating reports that were prepared by the vessel crew are recorded in the noon report. Thus, these two datasets were combined and for the specific purpose of this study, some features are chosen through the feature engineering step. The list of the features can be seen in Table 2.

The size of the original data set from the cargo vessel is 89,297 rows with a total of 151 columns, but only the 17 features in Table 2 were arbitrarily chosen for this analysis. These features are oceanographic and vessel maneuverability variables collected from the noon report. Oceanographic factors refer to the factors related to meteorology and (physical) oceanography, such as wind, wave, current, and sea surface measurements. Whereas maneuverability factors refer to the variables such as draft, rudder, ship heading, and speed. The target variable which is the shaft power was collected from the engine sensors data.

## 3. Research Methodologies

### 3.1. Machine Learning Prediction

In the process of developing a regression-based machine learning model to predict the vessel shaft power, several different approaches and ideas are put into practice to demonstrate that the attempt at model development was effective. In general, this research utilized a tree-based algorithm for several performance metrics. To begin the comprehensive procedure of choosing the regression model, we conducted a comparative analysis examining four different tree-based algorithms.

#### 3.1.1. Tree-Based Regressor Comparative Study

The tree-based technique we referred to in this study is an ensemble method that combines many decision trees to acquire higher prediction performance than a single decision tree. There are at least two methods that are widely used to develop tree ensembles, which are bagging and boosting [37]. There are numerous developed algorithms based on decision trees and ensemble formation. Among all, Random Forest [38] has proven to outperform Tree Bagging and other random tree ensemble methods [39]. Thus, Random Forest which is also one advanced tree-based method is utilized in this research. Random Forest combines the principle of bagging bootstrap sampling with extra randomization of the input attributes used as candidates to divide an inner node of the tree. Instead of attempting to find the best split among all features, the method randomly chooses a subset of features at each node and then calculates the best test over these features to effectively divide the node. This strategy is extremely effective and has found several successful applications in a variety of industries.

Aside from the bagging method, the decision tree-based algorithm also has its boosting categorization. Up to this day, there have been some gradient boosting algorithms that have been extended into some different methods, mentioned in order of the most recently developed, which are CatBoost (Category Boosting) [40], LightGBM (Light Gradient Boosted Machine) [41], and XGBoost (eXtreme Gradient Boosting) [42]. Focusing on processing speed and accuracy, all these gradients boosting-based techniques are deployed in the combination of weak learners into strong learners.

Additionally, it is proven by [43] that to get the best model parameters for a tree-based machine learning algorithm, among all the parameters, the learning rate and the depth of the trees can be optimized by performing hyperparameter tuning. Thus, this research utilized Grid Search Cross Validation (GridSearchCV) to perform the hyperparameter tuning and evaluate the models.

#### 3.1.2. Performance Evaluation

The model developed to predict the vessel shaft power was evaluated using metrics for regression-based machine learning models. These metrics provide the representation of the model’s error rate and accuracy. Among the existing metrics, this research used:

1. Mean Absolute Error (MAE)

Mean Absolute Error (MAE) is a model evaluation metric for regression models concerning its test set [44]. MAE measures the quality of fit in terms of the prediction error or the difference between the prediction results to the actual training data. It is calculated as:

(1) $$MAE=\frac{1}{m}\overset{m}{\underset{i=1}{\sum }}\left|X_{i}-Y_{i}\right|,$$

2. Root Mean Squared Error (RMSE)

RMSE emphasizes more on the larger absolute error of the model performance metrics.

(2) $$RMSE=\sqrt{\frac{1}{m}\overset{m}{\underset{i=1}{\sum }}{\left(X_{i}-Y_{i}\right)}^{2}},$$

The function has been widely adopted to standardize the unit measure of MSE [45].

(3) $$MSE=\frac{1}{m}\overset{m}{\underset{i=1}{\sum }}{\left(X_{i}-Y_{i}\right)}^{2},$$

MSE and RMSE are connected to one another in a way that is always calculated the same way through the square root. Both the MSE ordering and the RMSE ordering of the regression models will produce the exact same results.

3. Mean Absolute Percentage Error (MAPE)

Based on [46], MAPE has a consistent empirical risk minimization which represents a good basis to understand the limits of the machine learning algorithm. The calculated MAPE value shows the average deviation between the predicted value and the actual one.

(4) $$MAPE=\frac{1}{m}\overset{m}{\underset{i=1}{\sum }}\left|\frac{Y_{i}-X_{i}}{Y_{i}}\right|,$$

4. R^2^ or R-squared (Coefficient of Determination)

R-squared provides a more informative calculation of prediction performance metrics compared to MSE, RMSE, MAE, MAPE, and SMAPE [47]. According to [48], the coefficient of determination of R-squared is determined by calculating the proportion of the variance in the dependent variable that is predictable from the independent variable.

(5) $$R^{2}=1-\frac{\sum_{i=1}^{m}{\left(X_{i}-Y_{i}\right)}^{2}}{\sum_{i=1}^{m}{\left(\bar{Y}-Y_{i}\right)}^{2}},$$

### 3.2. Explainable Artificial Intelligence

As stated in the previous chapter, this research demonstrated a concept to interpret the black box of machine learning using Explainable Artificial Intelligence (XAI) methodology. There are several methodologies related to the implementation of XAI. One is categorized as heuristic explanations that are computed by approaches such as LIME [49], SHAP [50], or Anchor [51]. Among the three, this research implemented SHAP as it has the TreeExplainer method to specifically explain the output of the tree-based machine learning model.

**Figure 2:**
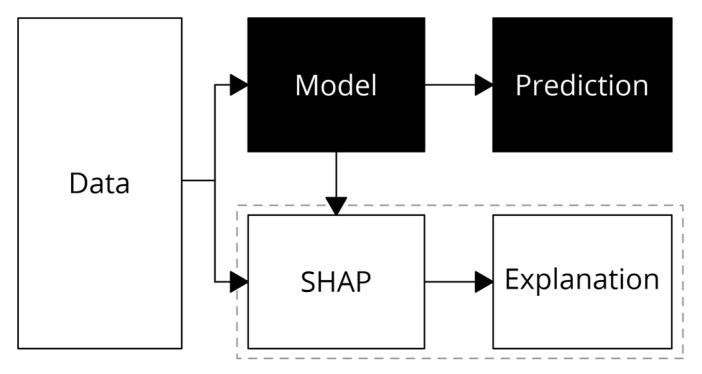
SHAP concept.

The basic concept of SHAP is shown in Figure 2. Firstly, a black-box model is trained from the data. Based on this trained model, SHAP has the role to produce a model explanation given the testing data. The explanation was presented in a form of Shapley values that calculated the feature contribution to the model output.

SHAP (Shapley Additive exPlanations) values are calculated using a method called the “Shapley value” introduced by Lloyd S. Shapley [52] which measures the fair allocation results of the cooperative game. Shapley values provide a way to fairly distribute a value among a group of individuals, where each individual’s contribution is based on their unique characteristics and the characteristics of the others in the group.

The basic idea behind SHAP values is to assign each feature an importance value for a given prediction. The method assigns a value of importance for each feature by considering all possible combinations of features and the marginal contribution of each feature to the prediction. Suppose that there is a set of input $X=\left\{x_{1},x_{2},I,x_{n}\right\}$ and a machine learning model v for every subset of the inputs, and S is the subset of X with the size of k(S), so that v(S) is the value of the subset. Then, the Shapley value for specific feature is estimated as the following:

(6) $$\varphi_{x}\left(v\right)=\frac{1}{n}\underset{s}{\sum }\frac{\left[v\left(S\cup \left\{x\right\}\right)-v\left(S\right)\right]}{\left(\begin{array}{c} n-1\\ k\left(S\right) \end{array}\right)},$$

where [v(S∪{x})−v(S)] is the marginal contribution of x for a given subset S. This calculation is repeated for all observations in the data set, resulting in a set of feature importance values for each observation. Once all the feature importance values are calculated, they can be used to interpret the importance of each feature for each observation in the data set. However, exact calculation of Shapely Value is computationally expensive because the size of feature permutation S increases exponentially with the number of features SHAP is a kind of an approximation for exact Shapely Value. Several types of SHAP model are proposed such as kernel SHAP, tree SHAP, or deep SHAP. To simplifies the calculation, each method assumes feature independence, or tries to exploit the structure of the black box model.

SHAP values provide a unified measure of feature importance that can be used for any model, regardless of whether it is a tree-based model, a linear model, or a neural network. SHAP values also have several attractive properties, such as being consistent with locally accurate feature importance measures and being able to consistently identify the feature importance of interaction effects. Correlation, on the other hand, only measures linear association between two variables, it does not take into account possible non-linear association, for example, SHAP values can detect feature importance when the correlation between input and output is zero. Also, correlation does not provide information of feature importance for a specific model or prediction.

In essence, local explanation typically relates to the process of explaining a single prediction result by breaking down each feature’s contribution represents by the Shapley value of one feature summed with other features’ contribution.

Global explanation, on the other hand, explains how features entirely contribute to the prediction result over entire data. Specifically for tree-based machine learning, Lundberg [53,54] developed what is called TreeExplainer to improve the interpretability of tree-based models like random forests, decision trees, and gradient-boosted trees. Originally [50], SHAP calculated the local explanation of one prediction as follows:

(7) $$\hat{y_{i}}=shap_{0}+shap\left(X_{1i}\right)+shap\left(X_{2i}\right)+\ldots +shap\left(X_{pi}\right)\;$$

The sum of all SHAP values is equal to the difference between the actual prediction value for observation *i* and the average prediction of overall data [55]. The model predicts the $\hat{y_{i}}$ by adding the $shap_{0}$, the mean prediction across all data, and the $shap\left(X_{ji}\right)$, which is the SHAP value for the *j*th feature for observation i that represents the marginal contribution of the feature to the model’s prediction, where in Equation (6) is the [v(S∪{x})−v(S)].

**Figure 3:**
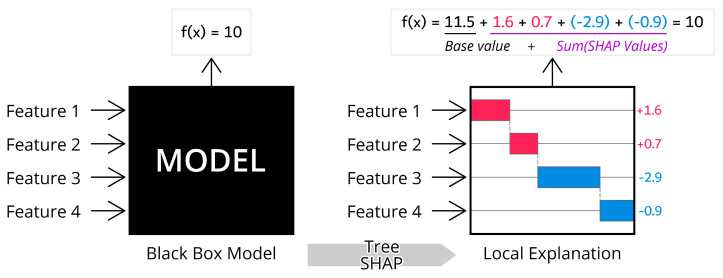
TreeSHAP concept.

Presented in Figure 3 is an illustrative example of how SHAP presented a local explanation of an individual prediction with feature attribution.

As shown, the individual prediction result of f(x)=10 can be decomposed with added contribution value (summation of Shapley values) of all features, which in Figure 3 is equal to 1.6+0.7−2.9−0.9=−1.5, to the model’s fixed base value, 11.5. In regression case, base value refers to the mean of the target variable over entire data points. Thus, the model output after the disclosure of contribution values would be the prediction base value added with the summation of features’ Shapley values. By this, we can quantify which feature mostly affects the prediction in that particular individual prediction. Then, the aggregation of all features attribution would provide the global explanation of the model [56].

**Figure 4:**
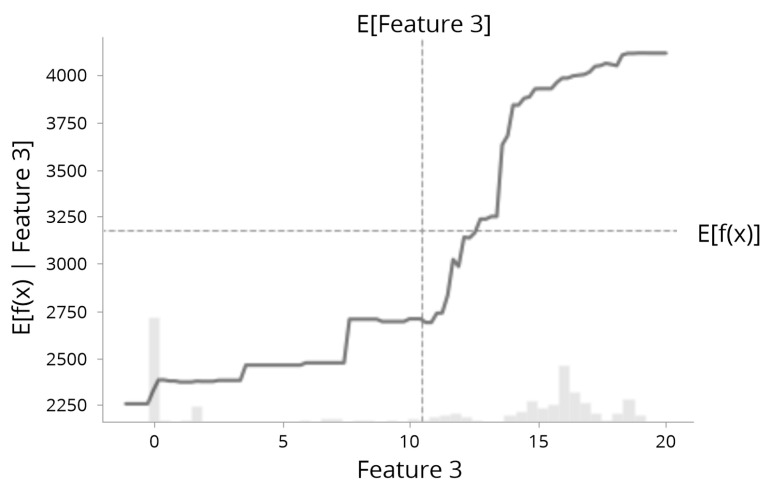
SHAP partial dependence plot illustration.

Moreover, to better understand the relationship between an individual feature’s value and the model’s prediction, Partial Dependence Plots (PDP) [57] can disclose the feature’s marginal contribution to the prediction result. The PDP functions can be used to interpret the results of any “black box” learning method [58]. In Figure 4, *X*-axis indicates the actual value of the feature complete with the histogram distribution of the data. The *Y*-axis is the expected prediction result given only a subset of ‘Feature 8′ is considered in the prediction.

As this research utilized tree-based machine learning, SHAP TreeExplainer [53] is implemented to explain the prediction model. TreeExplainer provides a fast and exact feature attribution method by exploiting an ensemble-based decision tree structure.

### 3.3. Experimental Framework

**Figure 5:**
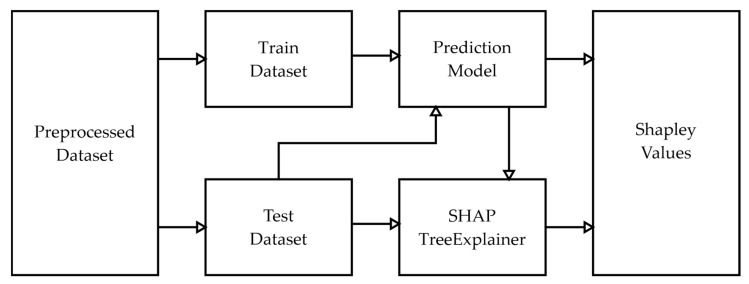
Experimental framework.

The general framework of this research is presented in Figure 5. The experiment began with the data preparation procedure that will be detailed in Section 3.1. Generally, this step consists of data preprocessing and feature engineering. As the final dataset is created, regression-based analysis was done to build a prediction model for shaft power by considering the oceanographic and maneuverability factors. Section 3.2. will explain the machine learning algorithm used in this step, that is Random Forest Regressor.

Continued to the next step, to explain the learning mechanism of the machine learning model that is built in the previous step, utilizing Shapley Additive exPlanations (SHAP) methodology, the black box of the machine learning is disclosed. The calculation of the Shapley value in this procedure is able to mathematically explain each feature’s contribution to the machine learning prediction result. The concept of the explainable artificial intelligence method utilized in this step has been presented in Section 3.2. The resulting Shapley values then being presented in some plots to better interpret the results.

## 4. Experimental Results and Discussion

This section includes a precise summary of the experimental results, interpretation, and research findings, which are presented in the sequence of data preprocessing, machine learning prediction, model explanation, and research discussion.

### 4.1. Data Preprocessing

It is necessary to process the dataset for it to be used in the experimental procedures. Consequently, in the stage of the study known as “data preprocessing,” and in this research, there are several primary stages that are carried out before the data is fed to the machine learning model. This research empirically constructed this sequence of procedures of data preprocessing specifically for the purpose of feeding a dataset to a shaft power prediction model with general machine learning algorithms. These procedures are as follows:

1. Features selection

In the direction to predict vessel shaft power in this research, from the 151 features included in the original dataset, only features related to the oceanographic and maneuverability of the vessel during its operational status are chosen as seen in the Table 3. There are two ways in deciding which features to choose. One is by expert judgment, which means deciding the chosen features based on maritime engineering practice. In the end, some features that are chosen include oceanographic information such as wave, wind, current, water depth, and sea surface profile during the vessel voyage, and maneuverability features such as vessel speed over ground, both of the drafts, and the rudder angle, as well as the target variable, the main engine shaft power.

2. Data filtering

Before the data is fed to the regression model that we have chosen based on the comparative study that we have conducted, we filtered the data based on domain expert percipience. First of all, when there is an error in the sensor systems, the system will record the data as −9999, and instances containing this value for each feature was removed. Seen in Table 4 below is the number of instances per feature that contain such error data and the ratio over the entire data.

Then, some specific feature values were filtered on their rational value range which is confirmed by the expert in maritime engineering. One is the total wave height which is no more than 6 m. Also, the speed over ground is set to be above 10 knots for it is indicated that the vessel is in cruise mode, as in it is working on the operational speed, sailing above the ocean.

From the chosen features, the statistical summary presented in Table 5 showed the distribution of the data numerically. Some indicators such as mean, maximum, and minimum value, as well as the standard deviation and variance of each feature, are presented.

3. Features transformation

The first transformation of features was between the draft fore and draft aft values. Both are values of vessel trim when the respective front and rear part of the hull from sea level, or in maritime engineering jargon, they are

(8) $$AvgDraft=\frac{Draft\;Fore+Draft\;Aft}{2}$$

Vertical distance between the waterline and the bottom of the hull measured at the perpendicular of the bow. Vertical distance between the waterline and the bottom of the hull measured at the perpendicular of the stern.

**Figure 6:**
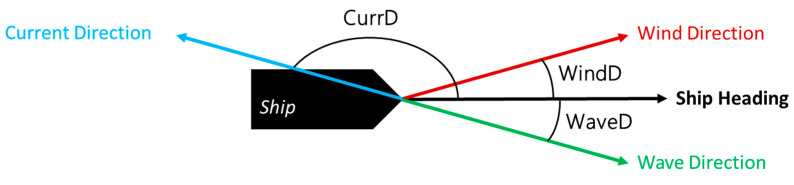
Directional feature transformation.

The dataset also has features indicating angular measurements, such as ship heading, rudder angle, wind and relative wind direction, current direction, and total wave direction. These features need to be transformed as they have different nature from the other magnitudes from features with scalar quantities. Their value generally ranged between 0° to 360° or −360° to 360°. As the vessel moves in the direction of the ship heading, all directional values except the ship heading are measured by the difference between the ship heading angle. This calculation intended to transform those directions into a scalar by calculating the angular difference between all directional features with the main direction of the vessel which is the ship heading angle and make some new scalar quantities. The transformation procedure is seen in Figure 6.

The final dataset after preprocessing then consists of 47,444 rows with 15 columns as seen in Table 6.

### 4.2. Prediction Results

Shaft power prediction was carried out by considering the oceanographic and maneuverability factors of the vessel as predictors. Utilizing a regression-based machine learning algorithm, this process is considered successful as the performance evaluation metrics showed a good result. To better choose the machine learning algorithm for our prediction model, we compared at least four advanced tree-based regression models, and the results were evaluated based on the performance metrics such as R-squared for the prediction performance measure, and error rate evaluation with RMSE, MAE, and MAPE.

The comparative analysis was also done by conducting hyperparameter tuning using Grid Search Cross-validation (GridSearchCV) with k = 5 or 5-folds cross-validation. Configuration of the parameters for the four algorithms that we compared that were tuned using GridSearchCV is shown in Table 7.

Furthermore, with the tuned parameters as the result of hyperparameter tuning using 5-fold GridSearchCV, the four models were predicting the shaft power with the entire dataset and the result is presented in Table 8. Comparing the four tree-based regressors was meant to find the best-performing model and as seen in Table 8, Random Forest Regressor has the best evaluation score. Thus, we chose Random Forest Regressor as the regression model.

Additionally, the analysis for the shaft power prediction was done in two separate queries. The first one was done using all data regardless of voyage number. So, all 47,444 instances were fed to the machine learning model, and four metrics to evaluate the prediction quality including R-squared score, RMSE, MAE, and MAPE were calculated. Then, with the same procedure, we conducted a separate analysis per distinct voyage number with the initial indication that different voyages may have each unique results because of the different oceanographic and maneuverability factors as the vessel sailed above the different oceans on different voyage numbers.

Therefore, to better examine this indication, performance evaluation with error calculation using RMSE, MAE, and MAPE, was also conducted over different voyage numbers. The complete results of the prediction analysis per voyage trip can be seen in Table 9. There is some prediction that has lower or higher performance measurement compared to the analysis over the entire data regardless of the voyage number. This fact alone can imply that on the different oceanographic and maneuverability factors, there must be changes in prediction performance.

Up to this, the result of the analysis has answered the initial research question No.1, that it is possible to predict the vessel shaft power considering the influence of uncontrollable variables such as the oceanographic factors and also the controllable variables such as the maneuverability factors. However, to pinpoint what factors are affecting the shaft power the most, the black box of the prediction model has to be disclosed. The Explainable Artificial Intelligence (XAI) approach is presented by utilizing the SHAP methodology to answer the next research question of which among the predictors affects the shaft power the most.

### 4.3. Explainable Machine Learning with SHAP

Previously, it is presented that with Random Forest Regressor, a prediction model was developed and predicted the shaft power of the overall dataset, as well as the dataset from each distinct voyage number. Then, the model and test data were fed to the explainable AI tools, SHAP, that were utilized in this research.

In SHAP methodology, the calculated Shapley values are presented in several types of visualization. In this research, a beeswarm type of summary plot is presented to show the overall feature importance of all features by the calculated Shapley values, and the averaged SHAP value over the entire data for every feature is presented using the mean absolute SHAP value bar plot. Then, deeper into the mathematical representation, the dependence plot and partial dependence plot will show how each feature (or at least the top 3 features) contributes to the model output.

**Figure 7:**
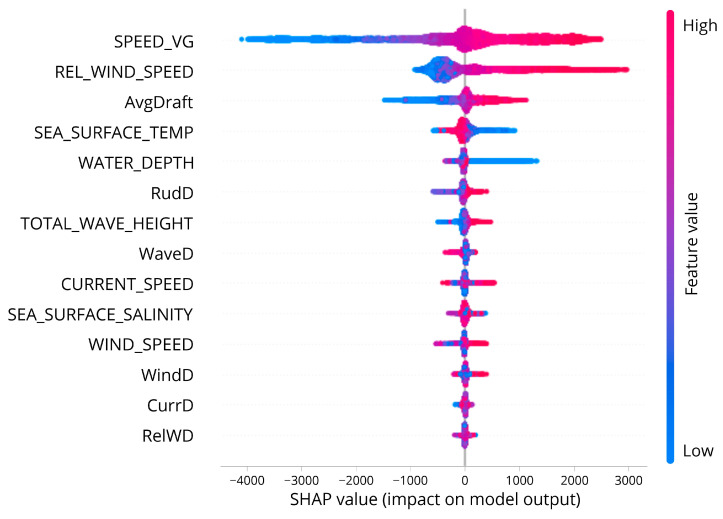
SHAP summary plot.

Presented in Figure 7, a summary plot visualized the Shapley values of each feature with respect to its impact on the model output along with the representation of each feature’s values. The color bar showed the real feature values where red indicates a higher value and blue otherwise. Each data point formed the horizontal plotting forming a violin shape. On the left side, the features are listed from top to the bottom in order of feature importance. Thus, SPEED_VG turned out to be the most important feature affecting the prediction of shaft power followed by REL_WIND_SPEED and AvgDraft.

**Figure 8:**
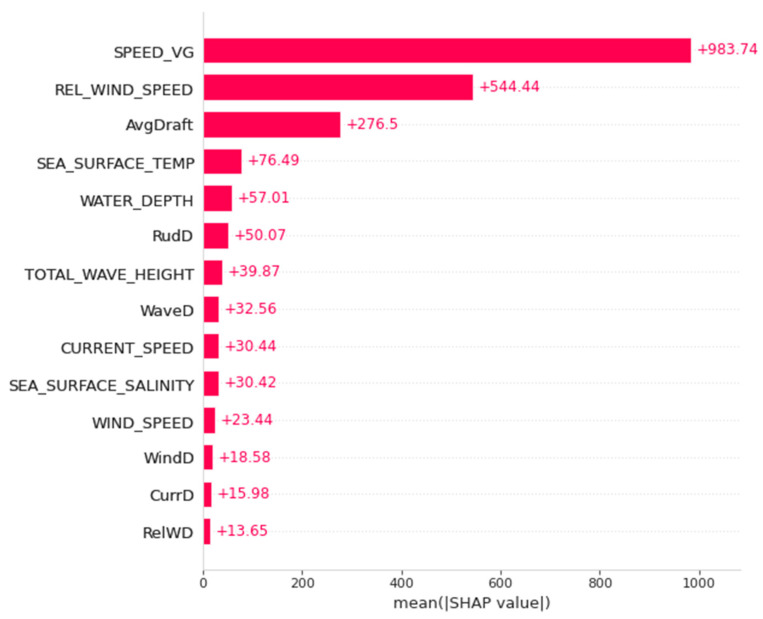
Mean absolute SHAP value bar plot.

Additionally, Figure 8 showed the mean absolute SHAP value for each feature that disclosed the average Shapley value of one predictor that contribute to the average prediction result. For example, for SPEED_VG, it is shown that for the average prediction result, or the base value, that is equal to 5540.4589 based on the SHAP calculation, the SPEED_VG averagely contributes to the 983.74 points of it.

These results drew the conclusion that, from the analysis of the prediction model towards the overall data of the 24 voyages, the speed over the ground of the vessel highly affects the prediction result. Simply, based on the SPEED_VG data fed into the model as the predictor, when the vessel moves at a faster speed over the ground, there will likely be bigger shaft power generated to move the propeller.

**Figure 9:**
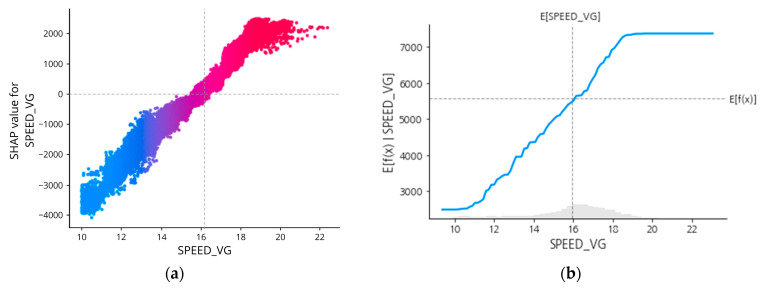
SHAP dependence plot (**a**) and partial dependence plot (**b**) of SPEED_VG.

**Figure 10:**
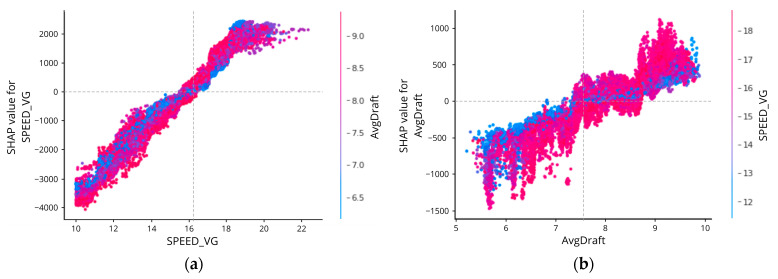
SHAP dependence plot of SPEED_VG with AvgDraft as interaction feature (**a**) and AvgDraft with SPEED_VG as interaction feature (**b**).

**Figure 11:**
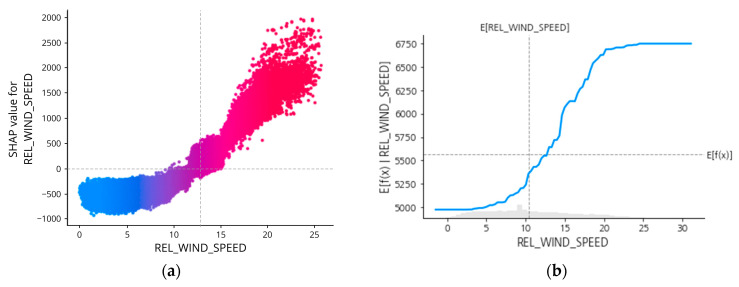
SHAP dependence plot (**a**) and partial dependence plot (**b**) of REL_WIND_SPEED.

**Figure 12:**
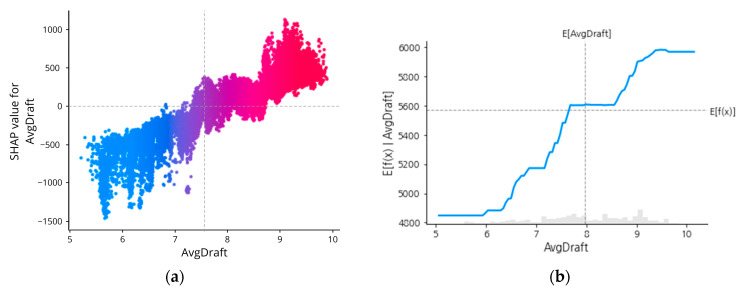
SHAP dependence plot (**a**) and partial dependence plot (**b**) of AvgDraft.

However, it is hard to tell the exact magnitude of SPEED_VG (or the other features) that affects the prediction results. To better understand this SHAP interpretation up to this, individualized attribution of the three most important features based on its Shapley Values are added respectively in Figure 9, Figure 10, Figure 11 and Figure 12, using a visualization called Dependence Plot (a) and Partial Dependence Plot (b).

Generally, the dependence plot showed the distribution of each feature’s Shapley value for each data instance and the red color represents high real values of the feature while blue is the opposite. Then, how the feature factual value impacts the prediction result can numerically be examined using the Partial Dependence Plot that shows the expected output of the prediction model.

First of all, as shown in Figure 9a, the dependence plot of feature SPEED_VG showed a constant tendency which is to have higher Shapley values given the higher feature value. Also, when the value falls below 16 knots, the SPEED_VG will be affecting the prediction result negatively or contributes a negative impact to the shaft power calculation as represented by the SHAP value line below 0.

To examine the attribution in a mathematical manner, Figure 9b showed the gray horizontal line in the plot that represents the expected value of the model when applied to the whole dataset. The vertical grey line represents the average value of the median feature value. So, for SPEED_VG, when the value is more than 16 knots, it will result in the expected value calculated for the target feature on the value above the horizontal line.

SPEED_VG is indeed the feature that is highly correlated with the target variable. It is a controllable variable as the value was controlled by the vessel operator along with other features like “AvgDraft” and “RudD” related to rudder angle. Thus, these controllable variables may have obvious feature attribution that is supposedly presented in the forms of the SHAP dependence plot and partial dependence plot.

If you see in Figure 10, the first and second figures showed the dependence plot of SPEED_VG and AvgDraft with interaction value. We can see how two features that are highly correlated contribute to the prediction result.

For REL_WIND_SPEED, is seen in Figure 11a that the positive impact of REL_WIND_SPEED on the prediction result happened when its value is relatively above 10.5 m/s.

The same manner can be practiced for the other feature as well, AvgDraft. The average draft the vessel maneuvered during its voyage affected the prediction results positively most likely when the draft of the ship (either draft aft or draft forward), showed the average draft above 8 will be more likely to increase the prediction results.

Additionally, this research also provided the analysis of vessel shaft power prediction over each distinct voyage number. Data were separated based on the recorded voyage number and a machine-learning model was run over each of the subsets.

**Figure 13:**
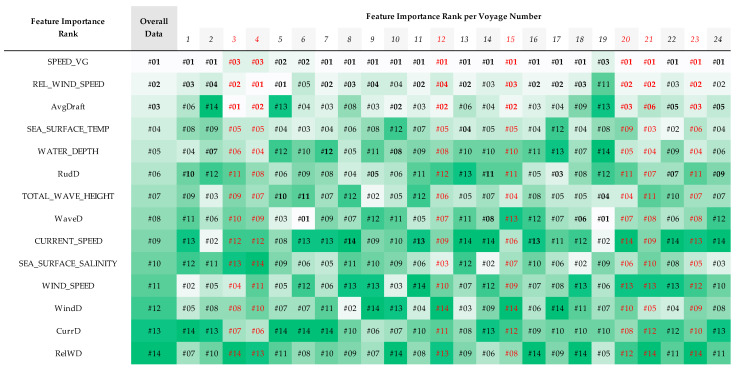
SHAP feature importance ranking heatmap.

Figure 13 showed the feature importance ranks based on the mean of Shapley values for each feature calculated by SHAP for analysis of all data and each distinct voyage number. Feature lists on the left side of the table showed sorted rank of feature importance overall data. Then, for the rest of the table, it presented the changes of rank for each respectable feature in the first column over each voyage number.

The top three features based on their average Shapley values representing their impact on the prediction result fluctuated in the terms of their feature importance ranks when the model analyze different voyage numbers.

## 5. Conclusions

Aligned with the initiative to reduce gas emissions overall transportation mode, as the major medium of transportation, the maritime industry has a big responsibility to the progression of the initiative. As one of the main measurements of a ship’s performance besides the speed, shaft power generated by the propulsion system motored by the vessel engine is analyzed in this research to find the best prediction model of this magnitude. This study began with three initial research questions.

1. Is it possible to predict the vessel shaft power considering the influence of uncontrollable variables such as the oceanographic factors and also the controllable variables such as the maneuverability factors?
2. Among the uncontrollable and controllable variables affecting the generation of vessel shaft power, what factors deploy the significant influence and how?
3. Does a different vessel voyage (trip) change the important factors affecting the vessel shaft power?

First of all, by considering oceanographic and maneuverability factors recorded during a real vessel voyage, utilizing Random Forest Regressor, this research reached a satisfactory result of prediction represented by the error rate and prediction accuracy. R-squared and MAPE of the prediction overall data showed 95% accuracy and 5% of error, while the average prediction accuracy of the analysis per distinct voyage number showed a higher score of 99% R-squared, while it resulted in a 5% of error on average. Thus, research question number 1 is attended.

Then, by implementing the SHAP approach, the model predicting the shaft power is explained by calculating each of the features of Shapley values. SHAP results feature the importance of model predictors, and the rank is presented in Figure 9, the SHAP Summary plot. Shown that the speed of the vessel (SPEED_VG) is the most affecting feature with respect to the overall prediction result, which is obvious because the generation of shaft power is linearly correlated with the generation of the speed of the ship. Comes as the second most affecting feature is one of the oceanographic factors, which is relative wind speed. Then, the third one is the average draft of the vessel. This answered question number 2.

For question number 3, it is proven that different voyage numbers or different vessel trips can change the feature importance of factors affecting the vessel shaft power. Certain features are seen to have stable ranks with respect to the Shapley value feature importance rank on different voyage numbers. However, the less important features are changing in terms of the impact of their value on the prediction results as represented by the fluctuated rank of the feature across different voyages.

Further work as the continuation of this research can consider a deeper analysis of what makes different vessel voyages affecting the result of SHAP feature importance. The indication is that different vessel voyages sailed above different sea profiles.

## Figures and Tables

**Table 1:** Vessel specifications.

Specification	Measurement
Length (m)	172.07
Beam (m)	27.4
Gross Tonnage	18,085
Deadweight (t)	22,317
TEU	1809
Year Build	2020

**Table 2:** Original feature lists.

Feature Name	Description
ME1_SHAFT_POWER	Propeller shaft power of the vessel
SPEED_VG	Speed over ground
DRAFT_FORE	Vertical distance between the waterline and the bottom of the hull measured at the perpendicular of the bow
DRAFT_AFT	Vertical distance between the waterline and the bottom of the hull measured at the perpendicular of the stern
SHIP_HEADING	Direction of which the vessel is sailing
TOTAL_WAVE_HEIGHT	Vertical distance between the crest (peak) and the trough of a wave
TOTAL_WAVE_DIRECTION	Direction of which the wave is moving
CURRENT_SPEED	Directional movement of seawater driven by gravity, wind (Coriolis Effect), and water density
CURRENT_DIRECTION	Direction of seawater
WIND_SPEED	Speed of the geographic or ground wind, assuming no tidal flow
WIND_DIRECTION	Direction of the geographic or ground wind
REL_WIND_SPEED	Speed of the apparent wind
REL_WIND_DIR	Direction of the apparent wind
RUDDER_ANGLE	The angle of the vessel rudder
WATER_DEPTH	The depth of water where the ship sails
SEA_SURFACE_SALINITY	Amount of salt on the body of the seawater
SEA_SURFACE_TEMP	Temperature on the seawater

**Table 3:** Features the summary statistics.

Feature Name	Min	Mean	Max	Standard Deviation
ME1_SHAFT_POWER	0	4072.26	9681.59	2920.40
SPEED_VG	−9999.00	11.4821	22.4	47.9097
REL_WIND_SPEED	−9999.00	7.4492	29.524	111.215
REL_WIND_DIR	−9999.00	184.9379	360	181.6887
DRAFT_FORE	2.633	7.5792	10.297	1.4717
DRAFT_AFT	5.707	8.1147	10.071	0.8196
SHIP_HEADING	−9999.00	144.976	359.998	135.9825
TOTAL_WAVE_HEIGHT	−9999.00	−141.738	8889.15	1188.97
TOTAL_WAVE_DIRECTION	−9999.00	5.8528	360	1208.92
CURRENT_SPEED	−9999.00	−142.8992	1.89	1187.64
CURRENT_DIRECTION	−9999.00	30.8615	360	1212.59
WIND_SPEED	−9999.00	−136.9188	27.25	1188.37
WIND_DIRECTION	−9999.00	5.4583	360	1209.35
RUDDER_ANGLE	−9999.00	−24.8728	36.8	512.351
WATER_DEPTH	−9999.00	−1341.97	834.6	3469.26
SEA_SURFACE_SALINITY	−9999.00	−111.0224	34.68	1191.48
SEA_SURFACE_TEMP	−9999.00	−120.9845	32.93	1190.31

**Table 4:** Error data counts.

Feature	Error Instances Count	Error Ratio (%)
ME1_SHAFT_POWER	0	0.00
SPEED_VG	2	0.00
DRAFT_FORE	0	0.00
DRAFT_AFT	0	0.00
SHIP_HEADING	7	0.01
TOTAL_WAVE_HEIGHT	1278	1.43
TOTAL_WAVE_DIRECTION	1278	1.43
CURRENT_SPEED	1278	1.43
CURRENT_DIRECTION	1278	1.43
WIND_SPEED	1278	1.43
WIND_DIRECTION	1278	1.43
RUDDER_ANGLE	235	0.26
SEA_SURFACE_SALINITY	1278	1.43
SEA_SURFACE_TEMP	1278	1.43
REL_WIND_SPEED	11	0.01
REL_WIND_DIR	11	0.01
WATER_DEPTH	12,352	13.83

**Table 5:** Features the summary statistics.

Feature Name	Min	Mean	Max	Standard Deviation
ME1_SHAFT_POWER	0	3810.3591	9681.594	2939.5802
SPEED_VG	0	11.0992	22.4	7.2974
REL_WIND_SPEED	0	8.5606	29.524	5.7281
REL_WIND_DIR	0	187.1479	360	141.6656
DRAFT_FORE	2.633	7.4488	10.297	1.4857
DRAFT_AFT	5.707	8.0631	10.071	0.8459
SHIP_HEADING	0	148.0478	359.998	102.9816
TOTAL_WAVE_HEIGHT	0.01	0.9648	5.97	0.8011
TOTAL_WAVE_DIRECTION	0.01	158.9932	360	91.5081
CURRENT_SPEED	0	0.184	1.89	0.1725
CURRENT_DIRECTION	0	176.6547	360	99.3439
WIND_SPEED	0.03	6.1868	27.25	3.6007
WIND_DIRECTION	0	156.57	360.00	97.79
RUDDER_ANGLE	−34.7	1.4153	36.8	1.8461
WATER_DEPTH	0.7	48.3948	834.6	75.1042
SEA_SURFACE_SALINITY	17.57	32.3563	34.68	2.3481
SEA_SURFACE_TEMP	−1.72	21.6276	32.62	8.2854

**Table 6:** Feature list after preprocessing.

Feature Name	Description
ME1_SHAFT_POWER	Propeller shaft power of the vessel
SPEED_VG	Speed over ground
TOTAL_WAVE_HEIGHT	Vertical distance between the crest (peak) and the trough of a wave
CURRENT_SPEED	Directional movement of seawater driven by gravity, wind (Coriolis Effect), and water density
WIND_SPEED	Speed of the geographic or ground wind, assuming no tidal flow
REL_WIND_SPEED	Speed of the apparent wind
WATER_DEPTH	The depth of water where the ship sails
SEA_SURFACE_SALINITY	Amount of salt on the body of the seawater
SEA_SURFACE_TEMP	Temperature on the seawater
AvgDraft ^1^	Averaged value of DRAFT_FORE and DRAFT_AFT
CurrD ^1^	Angular difference between SHIP_HEADING and CURRENT_DIRECTION
WaveD ^1^	Angular difference between SHIP_HEADING and TOTAL_WAVE_DIRECTION
WindD ^1^	Angular difference between SHIP_HEADING and WIND_DIRECTION
RelWD ^1^	Angular difference between SHIP_HEADING and REL_WIND_DIR
RudD ^1^	Angular difference between SHIP_HEADING and RUDDER_ANGLE

^1^ Transformed features.

**Table 7:** Hyperparameter tuning attribution.

Model	Parameters		
	Grid Parameters	Grid Values	Tuned Values
Random Forest Regressor	n_estimators max_depth min_samples_split min_samples_leaf	: [10,25,50,75,100] : [10,25,50,75,100] : [2,4,6,8,10] : [1,2,3,4,5]	{n_estimators: 100}, {max_depth: 25}, {min_samples_split: 2}, {min_samples_leaf: 1}
CatBoost Regressor	depth learning_rate iterations	: [10,25,50] : [0.1,0.5,1] : [50,100,250]	{depth: 10}, {learning_rate: 0.1}, {iterations: 250}
Extreme Gradient Boosting (XGB) Regressor	n_estimators max_depth learning_rate	: [50,100,150,200,250] : [5,10,25,50] : [0,0.5,1]	{n_estimators: 250}, {max_depth: 7}, {learning_rate: 0.01}
Light Gradient Boosting Machine (LightGBM) Regressor)	n_estimators max_depth learning_rate	: [50,75,100] : [10,50,100] : [0.05,0.1,0.5,1]	{n_estimators: 100}, {max_depth: 10}, {learning_rate: 0.5}

**Table 8:** Comparative performance measurements.

Model	R-Squared Score		RMSE		MAE		MAPE	
	Train	Test	Train	Test	Train	Test	Train	Test
Random Forest Regressor	0.99	0.95	156.95	414.88	82.76	221.00	0.02	0.05
CatBoost Regressor	0.98	0.95	237.69	393.73	164.66	248.59	0.04	0.06
Extreme Gradient Boosting (XGB) Regressor	1.00	0.94	2.84	446.56	1.88	263.60	0.00	0.06
Light Gradient Boosting Machine (LightGBM) Regressor)	0.98	0.94	221.95	449.48	159.27	289.12	0.03	0.07
Average Scoring	0.99	0.95	154.86	426.16	102.14	255.58	0.02	0.06

**Table 9:** Performance metrics per voyage number.

VOY.NO	#Instances	R-Squared Score		RMSE		MAE		MAPE	
		Train	Test	Train	Test	Train	Test	Train	Test
1	522	0.98	0.93	105.85	210.44	37.70	77.92	0.02	0.03
2	1852	0.99	0.95	115.49	302.66	58.23	146.60	0.02	0.04
3	2391	0.99	0.92	157.32	502.26	77.55	228.38	0.02	0.06
4	2367	0.99	0.92	158.76	432.59	75.43	201.87	0.02	0.05
5	2192	0.99	0.93	163.04	384.86	67.86	166.49	0.02	0.04
6	2258	0.99	0.92	131.93	351.80	58.13	155.41	0.02	0.04
7	2260	0.98	0.90	164.63	313.51	65.74	138.47	0.02	0.05
8	2458	0.97	0.79	156.69	391.22	62.43	160.65	0.02	0.05
9	1796	0.98	0.85	147.04	420.97	58.71	164.66	0.02	0.05
10	2112	0.99	0.94	131.45	339.46	58.26	148.70	0.02	0.04
11	2183	0.99	0.93	199.58	484.96	90.11	219.33	0.03	0.06
12	2064	0.99	0.94	134.09	419.91	68.52	209.99	0.02	0.06
13	1641	0.99	0.95	179.03	353.58	73.79	169.67	0.02	0.04
14	2253	0.99	0.93	150.80	376.11	61.88	179.48	0.02	0.05
15	2418	0.99	0.95	177.57	492.00	88.89	238.77	0.02	0.05
16	2065	0.99	0.93	183.39	517.22	99.79	285.36	0.02	0.07
17	2063	1.00	0.96	160.98	421.94	75.34	213.15	0.02	0.06
18	1877	0.99	0.96	169.40	448.25	79.10	214.77	0.02	0.05
19	560	0.99	0.93	147.37	328.36	74.77	194.51	0.01	0.03
20	2041	0.99	0.96	160.51	459.91	78.96	213.19	0.02	0.05
21	1967	1.00	0.96	169.01	456.15	68.84	195.63	0.02	0.06
22	1969	0.99	0.94	162.54	426.96	76.08	198.78	0.02	0.05
23	2773	0.99	0.95	164.23	402.20	74.28	189.41	0.02	0.05
24	1362	0.99	0.96	149.60	391.09	107.18	216.51	0.03	0.07
Average per Voyage	1977	0.99	0.93	155.85	401.18	72.40	188.65	0.02	0.05
All Data	47,444	0.99	0.95	156.95	414.88	82.76	221.00	0.02	0.05
